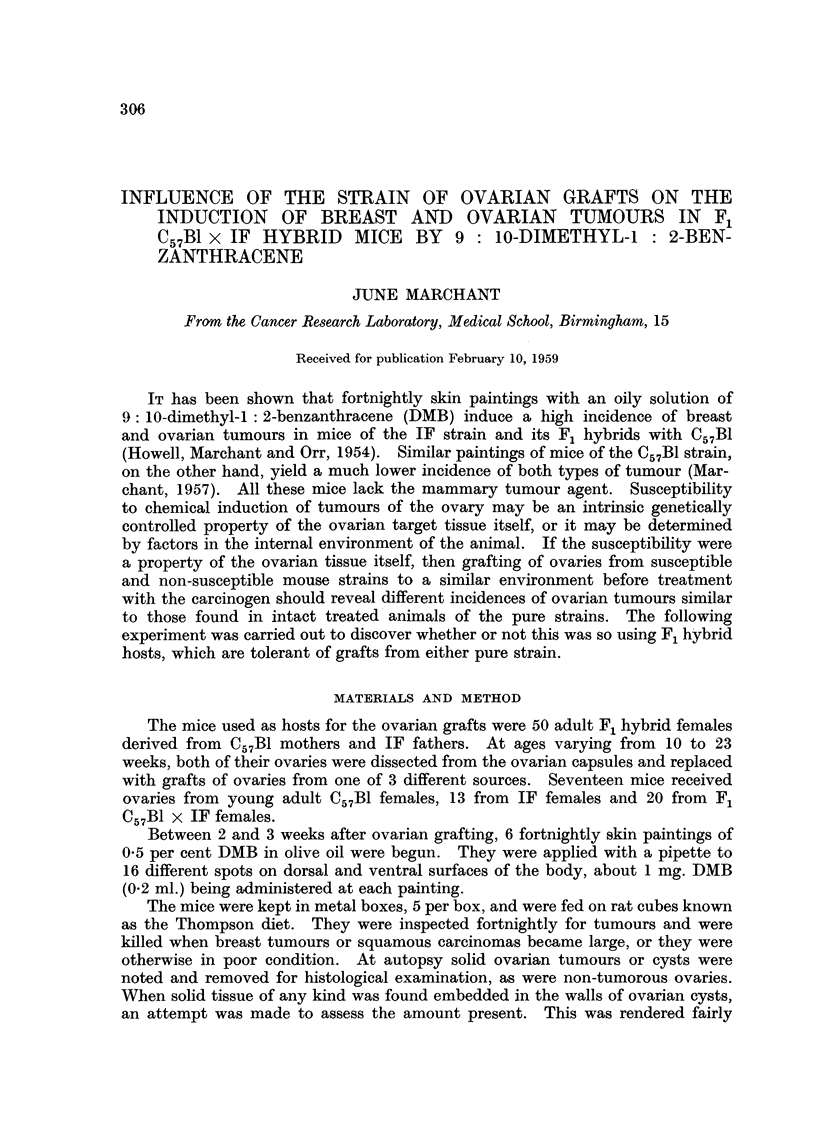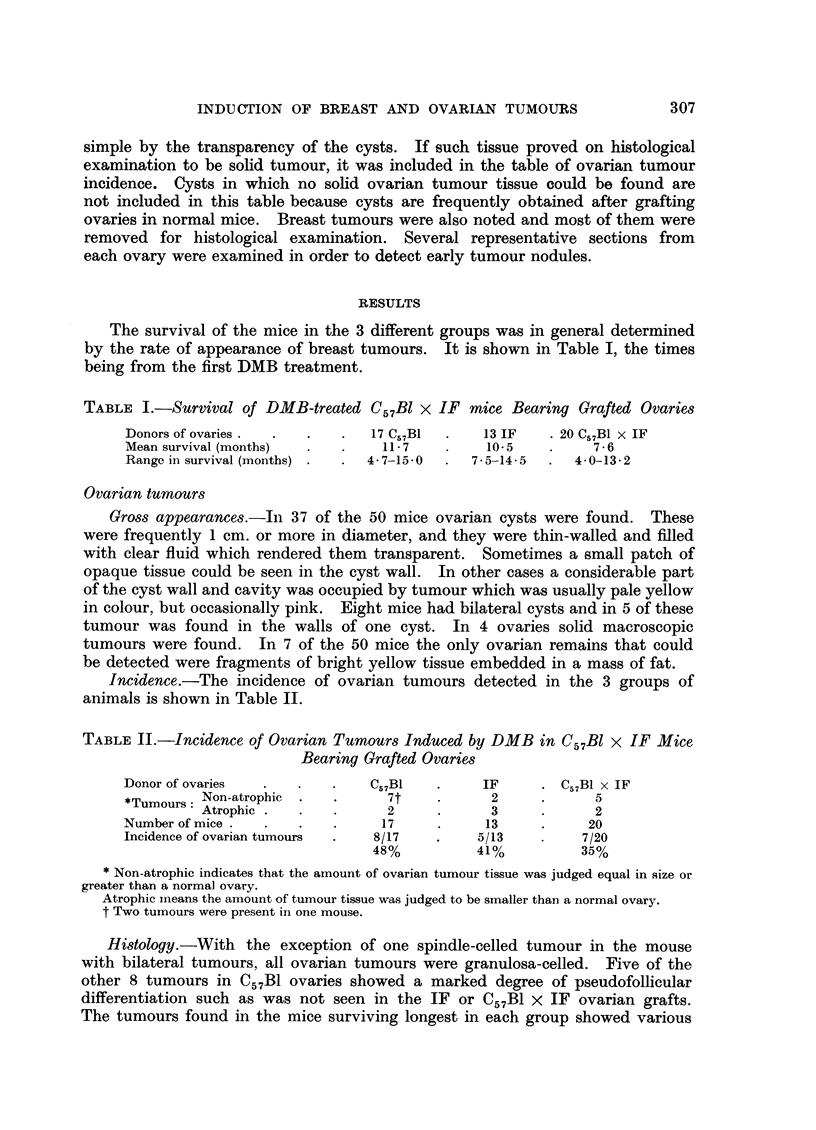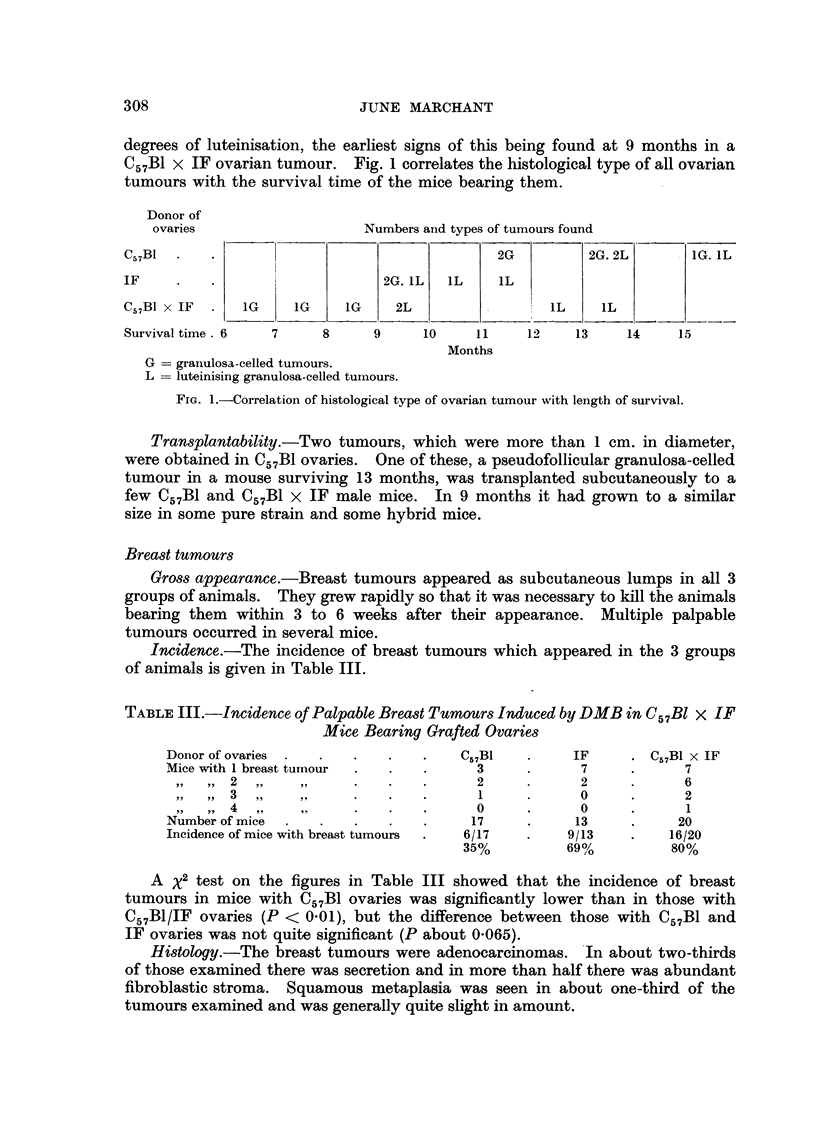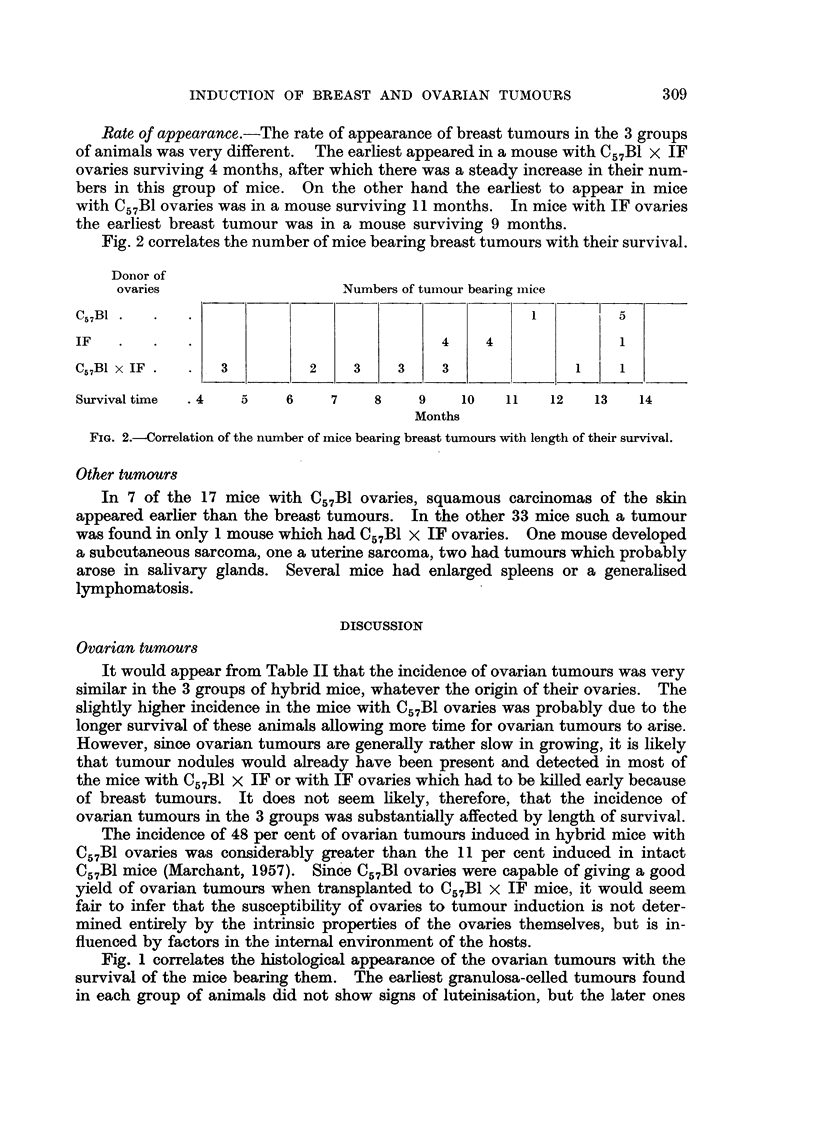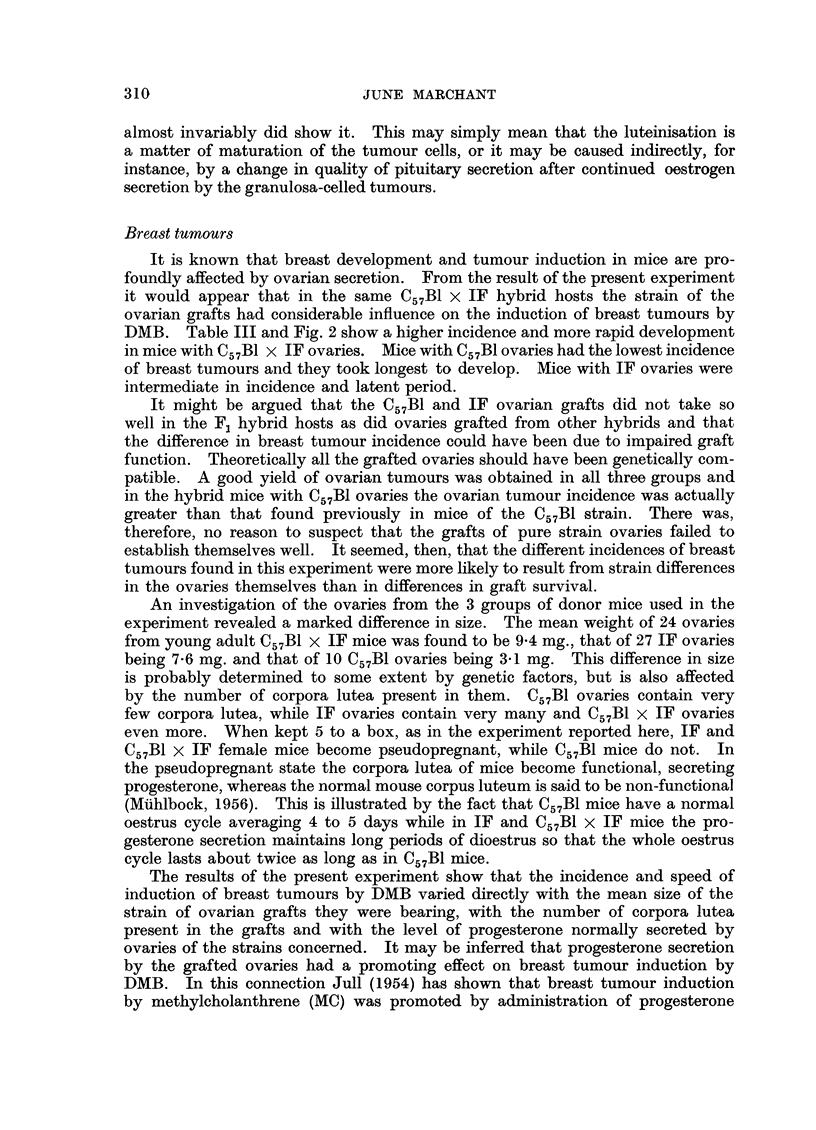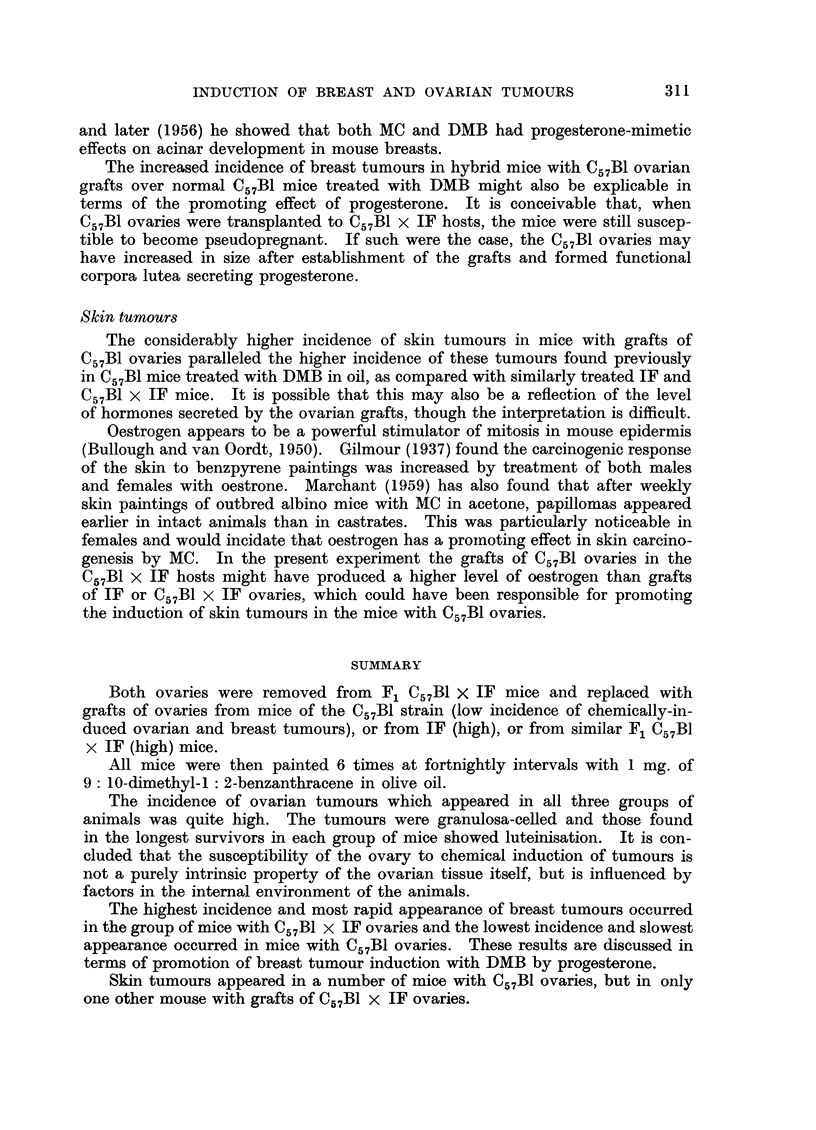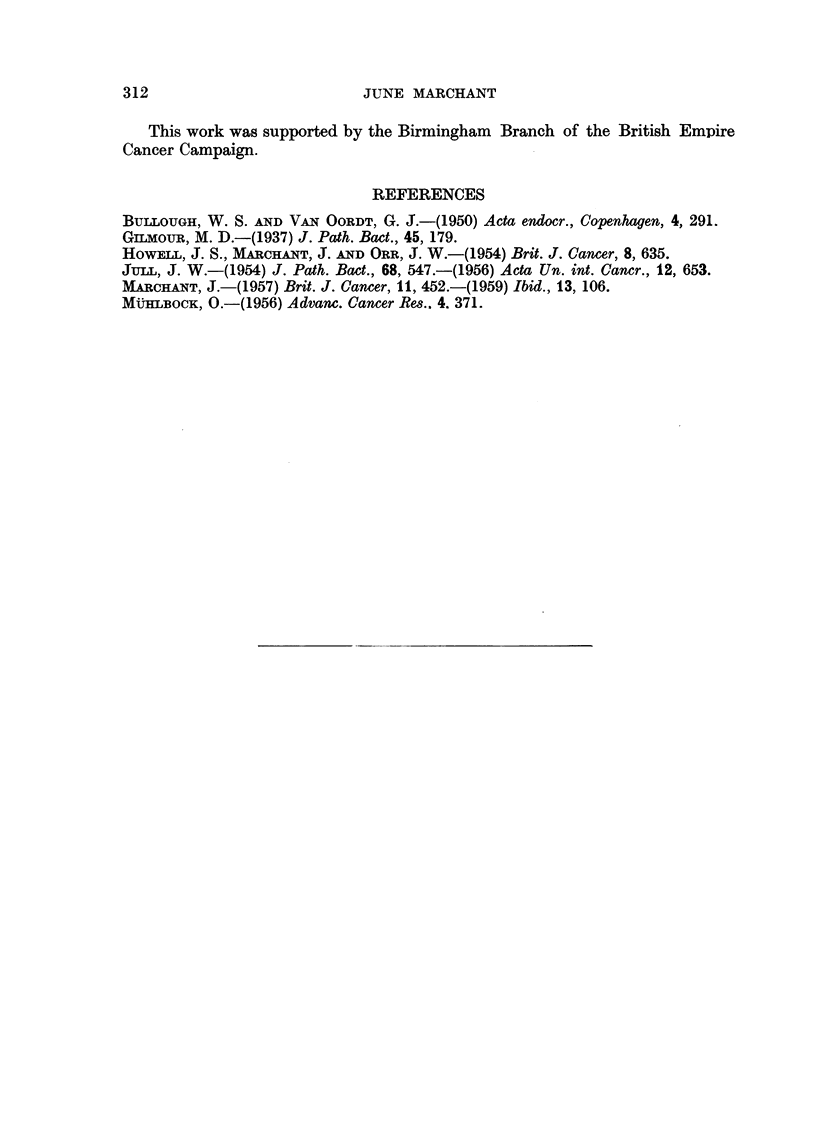# Influence of the Strain of Ovarian Grafts on the Induction of Breast and Ovarian Tumours in F_1_ C_57_B1 x IF Hybrid Mice by 9:10-Dimethyl-1:2-Benzanthracene

**DOI:** 10.1038/bjc.1959.37

**Published:** 1959-06

**Authors:** June Marchant


### INFLUENCE OF THE STRAIN OF OVARIAN GRAFTS ON THE INDUCTION OF BREAST AND OVARIAN TUMOURS IN $F_1$ $C_{57}Bl \times$ IF HYBRID MICE BY 9 : 10-DIMETHYL-1 : 2-BENZANTHRACENE

JUNE MARCHANT

*From the Cancer Research Laboratory, Medical School, Birmingham,* 15

Received for publication February 10, 1959

It has been shown that fortnightly skin paintings with an oily solution of 9 : 10-dimethyl-1 : 2-benzanthracene (DMB) induce a high incidence of breast and ovarian tumours in mice of the IF strain and its $F_1$ hybrids with $C_{57}Bl$ (Howell, Marchant and Orr, 1954). Similar paintings of mice of the $C_{57}Bl$ strain, on the other hand, yield a much lower incidence of both types of tumour (Marchant, 1957). All these mice lack the mammary tumour agent. Susceptibility to chemical induction of tumours of the ovary may be an intrinsic genetically controlled property of the ovarian target tissue itself, or it may be determined by factors in the internal environment of the animal. If the susceptibility were a property of the ovarian tissue itself, then grafting of ovaries from susceptible and non-susceptible mouse strains to a similar environment before treatment with the carcinogen should reveal different incidences of ovarian tumours similar to those found in intact treated animals of the pure strains. The following experiment was carried out to discover whether or not this was so using $F_1$ hybrid hosts, which are tolerant of grafts from either pure strain.

### MATERIALS AND METHOD

The mice used as hosts for the ovarian grafts were 50 adult $F_1$ hybrid females derived from $C_{57}Bl$ mothers and IF fathers. At ages varying from 10 to 23 weeks, both of their ovaries were dissected from the ovarian capsules and replaced with grafts of ovaries from one of 3 different sources. Seventeen mice received ovaries from young adult $C_{57}Bl$ females, 13 from IF females and 20 from $F_1$ $C_{57}Bl \times$ IF females.

Between 2 and 3 weeks after ovarian grafting, 6 fortnightly skin paintings of 0·5 per cent DMB in olive oil were begun. They were applied with a pipette to 16 different spots on dorsal and ventral surfaces of the body, about 1 mg. DMB (0·2 ml.) being administered at each painting.

The mice were kept in metal boxes, 5 per box, and were fed on rat cubes known as the Thompson diet. They were inspected fortnightly for tumours and were killed when breast tumours or squamous carcinomas became large, or they were otherwise in poor condition. At autopsy solid ovarian tumours or cysts were noted and removed for histological examination, as were non-tumorous ovaries. When solid tissue of any kind was found embedded in the walls of ovarian cysts, an attempt was made to assess the amount present. This was rendered fairly simple by the transparency of the cysts. If such tissue proved on histological examination to be solid tumour, it was included in the table of ovarian tumour incidence. Cysts in which no solid ovarian tumour tissue could be found are not included in this table because cysts are frequently obtained after grafting ovaries in normal mice. Breast tumours were also noted and most of them were removed for histological examination. Several representative sections from each ovary were examined in order to detect early tumour nodules.

### RESULTS

The survival of the mice in the 3 different groups was in general determined by the rate of appearance of breast tumours. It is shown in Table I, the times being from the first DMB treatment.

TABLE I.—*Survival of DMB-treated $C_{57}Bl \times IF$ mice Bearing Grafted Ovaries*

| | | | |
|---|---|---|---|
| Donors of ovaries | 17 $C_{57}$Bl | 13 IF | 20 $C_{57}$Bl × IF |
| Mean survival (months) | 11·7 | 10·5 | 7·6 |
| Range in survival (months) | 4·7–15·0 | 7·5–14·5 | 4·0–13·2 |

### *Ovarian tumours*

*Gross appearances.*—In 37 of the 50 mice ovarian cysts were found. These were frequently 1 cm. or more in diameter, and they were thin-walled and filled with clear fluid which rendered them transparent. Sometimes a small patch of opaque tissue could be seen in the cyst wall. In other cases a considerable part of the cyst wall and cavity was occupied by tumour which was usually pale yellow in colour, but occasionally pink. Eight mice had bilateral cysts and in 5 of these tumour was found in the walls of one cyst. In 4 ovaries solid macroscopic tumours were found. In 7 of the 50 mice the only ovarian remains that could be detected were fragments of bright yellow tissue embedded in a mass of fat.

*Incidence.*—The incidence of ovarian tumours detected in the 3 groups of animals is shown in Table II.

TABLE II.—*Incidence of Ovarian Tumours Induced by DMB in $C_{57}Bl \times IF$ Mice Bearing Grafted Ovaries*

| | | | |
|---|---|---|---|
| Donor of ovaries | $C_{57}$Bl | IF | $C_{57}$Bl × IF |
| *Tumours : Non-atrophic | 7† | 2 | 5 |
| *Tumours : Atrophic | 2 | 3 | 2 |
| Number of mice | 17 | 13 | 20 |
| Incidence of ovarian tumours | 8/17 | 5/13 | 7/20 |
| | 48% | 41% | 35% |

* Non-atrophic indicates that the amount of ovarian tumour tissue was judged equal in size or greater than a normal ovary.

Atrophic means the amount of tumour tissue was judged to be smaller than a normal ovary.

† Two tumours were present in one mouse.

*Histology.*—With the exception of one spindle-celled tumour in the mouse with bilateral tumours, all ovarian tumours were granulosa-celled. Five of the other 8 tumours in $C_{57}$Bl ovaries showed a marked degree of pseudofollicular differentiation such as was not seen in the IF or $C_{57}Bl \times IF$ ovarian grafts. The tumours found in the mice surviving longest in each group showed various degrees of luteinisation, the earliest signs of this being found at 9 months in a $C_{57}Bl \times IF$ ovarian tumour. Fig. 1 correlates the histological type of all ovarian tumours with the survival time of the mice bearing them.

| Donor of ovaries | Numbers and types of tumours found | | | | | | | | | |
|---|---|---|---|---|---|---|---|---|---|---|
| $C_{57}Bl$ . . | | | | | | 2G | | 2G. 2L | | 1G. 1L |
| IF . . | | | | 2G. 1L | 1L | 1L | | | | |
| $C_{57}Bl \times IF$ . | 1G | 1G | 1G | 2L | | | 1L | 1L | | |
| Survival time . | 6 | 7 | 8 | 9 | 10 | 11 | 12 | 13 | 14 | 15 |

Months

G = granulosa-celled tumours.
L = luteinising granulosa-celled tumours.

FIG. 1.—Correlation of histological type of ovarian tumour with length of survival.

*Transplantability.*—Two tumours, which were more than 1 cm. in diameter, were obtained in $C_{57}Bl$ ovaries. One of these, a pseudofollicular granulosa-celled tumour in a mouse surviving 13 months, was transplanted subcutaneously to a few $C_{57}Bl$ and $C_{57}Bl \times IF$ male mice. In 9 months it had grown to a similar size in some pure strain and some hybrid mice.

### *Breast tumours*

*Gross appearance.*—Breast tumours appeared as subcutaneous lumps in all 3 groups of animals. They grew rapidly so that it was necessary to kill the animals bearing them within 3 to 6 weeks after their appearance. Multiple palpable tumours occurred in several mice.

*Incidence.*—The incidence of breast tumours which appeared in the 3 groups of animals is given in Table III.

TABLE III.—*Incidence of Palpable Breast Tumours Induced by DMB in $C_{57}Bl \times IF$ Mice Bearing Grafted Ovaries*

| Donor of ovaries . . . . . . | $C_{57}Bl$ | IF | $C_{57}Bl \times IF$ |
|---|---|---|---|
| Mice with 1 breast tumour . . . | 3 | 7 | 7 |
| ,, ,, 2 ,, ,, . . . | 2 | 2 | 6 |
| ,, ,, 3 ,, ,, . . . | 1 | 0 | 2 |
| ,, ,, 4 ,, ,, . . . | 0 | 0 | 1 |
| Number of mice . . . . . | 17 | 13 | 20 |
| Incidence of mice with breast tumours . | 6/17 | 9/13 | 16/20 |
| | 35% | 69% | 80% |

A $\chi^2$ test on the figures in Table III showed that the incidence of breast tumours in mice with $C_{57}Bl$ ovaries was significantly lower than in those with $C_{57}Bl/IF$ ovaries ($P < 0{\cdot}01$), but the difference between those with $C_{57}Bl$ and IF ovaries was not quite significant ($P$ about 0·065).

*Histology.*—The breast tumours were adenocarcinomas. In about two-thirds of those examined there was secretion and in more than half there was abundant fibroblastic stroma. Squamous metaplasia was seen in about one-third of the tumours examined and was generally quite slight in amount.

*Rate of appearance.*—The rate of appearance of breast tumours in the 3 groups of animals was very different. The earliest appeared in a mouse with $C_{57}Bl \times IF$ ovaries surviving 4 months, after which there was a steady increase in their numbers in this group of mice. On the other hand the earliest to appear in mice with $C_{57}Bl$ ovaries was in a mouse surviving 11 months. In mice with IF ovaries the earliest breast tumour was in a mouse surviving 9 months.

Fig. 2 correlates the number of mice bearing breast tumours with their survival.

| Donor of ovaries | Numbers of tumour bearing mice | | | | | | | | | | |
|---|---|---|---|---|---|---|---|---|---|---|---|
| $C_{57}Bl$ . . . | | | | | | | | 1 | | 5 | |
| IF . . . | | | | | | 4 | 4 | | | 1 | |
| $C_{57}Bl \times IF$ . . | 3 | | 2 | 3 | 3 | 3 | | | 1 | 1 | |

Survival time . 4 5 6 7 8 9 10 11 12 13 14
Months

FIG. 2.—Correlation of the number of mice bearing breast tumours with length of their survival.

### *Other tumours*

In 7 of the 17 mice with $C_{57}Bl$ ovaries, squamous carcinomas of the skin appeared earlier than the breast tumours. In the other 33 mice such a tumour was found in only 1 mouse which had $C_{57}Bl \times IF$ ovaries. One mouse developed a subcutaneous sarcoma, one a uterine sarcoma, two had tumours which probably arose in salivary glands. Several mice had enlarged spleens or a generalised lymphomatosis.

### DISCUSSION

### *Ovarian tumours*

It would appear from Table II that the incidence of ovarian tumours was very similar in the 3 groups of hybrid mice, whatever the origin of their ovaries. The slightly higher incidence in the mice with $C_{57}Bl$ ovaries was probably due to the longer survival of these animals allowing more time for ovarian tumours to arise. However, since ovarian tumours are generally rather slow in growing, it is likely that tumour nodules would already have been present and detected in most of the mice with $C_{57}Bl \times IF$ or with IF ovaries which had to be killed early because of breast tumours. It does not seem likely, therefore, that the incidence of ovarian tumours in the 3 groups was substantially affected by length of survival.

The incidence of 48 per cent of ovarian tumours induced in hybrid mice with $C_{57}Bl$ ovaries was considerably greater than the 11 per cent induced in intact $C_{57}Bl$ mice (Marchant, 1957). Since $C_{57}Bl$ ovaries were capable of giving a good yield of ovarian tumours when transplanted to $C_{57}Bl \times IF$ mice, it would seem fair to infer that the susceptibility of ovaries to tumour induction is not determined entirely by the intrinsic properties of the ovaries themselves, but is influenced by factors in the internal environment of the hosts.

Fig. 1 correlates the histological appearance of the ovarian tumours with the survival of the mice bearing them. The earliest granulosa-celled tumours found in each group of animals did not show signs of luteinisation, but the later ones almost invariably did show it. This may simply mean that the luteinisation is a matter of maturation of the tumour cells, or it may be caused indirectly, for instance, by a change in quality of pituitary secretion after continued oestrogen secretion by the granulosa-celled tumours.

*Breast tumours*

It is known that breast development and tumour induction in mice are profoundly affected by ovarian secretion. From the result of the present experiment it would appear that in the same $C_{57}$Bl × IF hybrid hosts the strain of the ovarian grafts had considerable influence on the induction of breast tumours by DMB. Table III and Fig. 2 show a higher incidence and more rapid development in mice with $C_{57}$Bl × IF ovaries. Mice with $C_{57}$Bl ovaries had the lowest incidence of breast tumours and they took longest to develop. Mice with IF ovaries were intermediate in incidence and latent period.

It might be argued that the $C_{57}$Bl and IF ovarian grafts did not take so well in the $F_1$ hybrid hosts as did ovaries grafted from other hybrids and that the difference in breast tumour incidence could have been due to impaired graft function. Theoretically all the grafted ovaries should have been genetically compatible. A good yield of ovarian tumours was obtained in all three groups and in the hybrid mice with $C_{57}$Bl ovaries the ovarian tumour incidence was actually greater than that found previously in mice of the $C_{57}$Bl strain. There was, therefore, no reason to suspect that the grafts of pure strain ovaries failed to establish themselves well. It seemed, then, that the different incidences of breast tumours found in this experiment were more likely to result from strain differences in the ovaries themselves than in differences in graft survival.

An investigation of the ovaries from the 3 groups of donor mice used in the experiment revealed a marked difference in size. The mean weight of 24 ovaries from young adult $C_{57}$Bl × IF mice was found to be 9·4 mg., that of 27 IF ovaries being 7·6 mg. and that of 10 $C_{57}$Bl ovaries being 3·1 mg. This difference in size is probably determined to some extent by genetic factors, but is also affected by the number of corpora lutea present in them. $C_{57}$Bl ovaries contain very few corpora lutea, while IF ovaries contain very many and $C_{57}$Bl × IF ovaries even more. When kept 5 to a box, as in the experiment reported here, IF and $C_{57}$Bl × IF female mice become pseudopregnant, while $C_{57}$Bl mice do not. In the pseudopregnant state the corpora lutea of mice become functional, secreting progesterone, whereas the normal mouse corpus luteum is said to be non-functional (Mühlbock, 1956). This is illustrated by the fact that $C_{57}$Bl mice have a normal oestrus cycle averaging 4 to 5 days while in IF and $C_{57}$Bl × IF mice the progesterone secretion maintains long periods of dioestrus so that the whole oestrus cycle lasts about twice as long as in $C_{57}$Bl mice.

The results of the present experiment show that the incidence and speed of induction of breast tumours by DMB varied directly with the mean size of the strain of ovarian grafts they were bearing, with the number of corpora lutea present in the grafts and with the level of progesterone normally secreted by ovaries of the strains concerned. It may be inferred that progesterone secretion by the grafted ovaries had a promoting effect on breast tumour induction by DMB. In this connection Jull (1954) has shown that breast tumour induction by methylcholanthrene (MC) was promoted by administration of progesterone and later (1956) he showed that both MC and DMB had progesterone-mimetic effects on acinar development in mouse breasts.

The increased incidence of breast tumours in hybrid mice with $C_{57}$Bl ovarian grafts over normal $C_{57}$Bl mice treated with DMB might also be explicable in terms of the promoting effect of progesterone. It is conceivable that, when $C_{57}$Bl ovaries were transplanted to $C_{57}$Bl $\times$ IF hosts, the mice were still susceptible to become pseudopregnant. If such were the case, the $C_{57}$Bl ovaries may have increased in size after establishment of the grafts and formed functional corpora lutea secreting progesterone.

*Skin tumours*

The considerably higher incidence of skin tumours in mice with grafts of $C_{57}$Bl ovaries paralleled the higher incidence of these tumours found previously in $C_{57}$Bl mice treated with DMB in oil, as compared with similarly treated IF and $C_{57}$Bl $\times$ IF mice. It is possible that this may also be a reflection of the level of hormones secreted by the ovarian grafts, though the interpretation is difficult.

Oestrogen appears to be a powerful stimulator of mitosis in mouse epidermis (Bullough and van Oordt, 1950). Gilmour (1937) found the carcinogenic response of the skin to benzpyrene paintings was increased by treatment of both males and females with oestrone. Marchant (1959) has also found that after weekly skin paintings of outbred albino mice with MC in acetone, papillomas appeared earlier in intact animals than in castrates. This was particularly noticeable in females and would incidate that oestrogen has a promoting effect in skin carcinogenesis by MC. In the present experiment the grafts of $C_{57}$Bl ovaries in the $C_{57}$Bl $\times$ IF hosts might have produced a higher level of oestrogen than grafts of IF or $C_{57}$Bl $\times$ IF ovaries, which could have been responsible for promoting the induction of skin tumours in the mice with $C_{57}$Bl ovaries.

### SUMMARY

Both ovaries were removed from $F_1$ $C_{57}$Bl $\times$ IF mice and replaced with grafts of ovaries from mice of the $C_{57}$Bl strain (low incidence of chemically-induced ovarian and breast tumours), or from IF (high), or from similar $F_1$ $C_{57}$Bl $\times$ IF (high) mice.

All mice were then painted 6 times at fortnightly intervals with 1 mg. of 9 : 10-dimethyl-1 : 2-benzanthracene in olive oil.

The incidence of ovarian tumours which appeared in all three groups of animals was quite high. The tumours were granulosa-celled and those found in the longest survivors in each group of mice showed luteinisation. It is concluded that the susceptibility of the ovary to chemical induction of tumours is not a purely intrinsic property of the ovarian tissue itself, but is influenced by factors in the internal environment of the animals.

The highest incidence and most rapid appearance of breast tumours occurred in the group of mice with $C_{57}$Bl $\times$ IF ovaries and the lowest incidence and slowest appearance occurred in mice with $C_{57}$Bl ovaries. These results are discussed in terms of promotion of breast tumour induction with DMB by progesterone.

Skin tumours appeared in a number of mice with $C_{57}$Bl ovaries, but in only one other mouse with grafts of $C_{57}$Bl $\times$ IF ovaries.

This work was supported by the Birmingham Branch of the British Empire Cancer Campaign.

### REFERENCES

BULLOUGH, W. S. AND VAN OORDT, G. J.—(1950) *Acta endocr., Copenhagen*, **4**, 291.
GILMOUR, M. D.—(1937) *J. Path. Bact.*, **45**, 179.
HOWELL, J. S., MARCHANT, J. AND ORR, J. W.—(1954) *Brit. J. Cancer*, **8**, 635.
JULL, J. W.—(1954) *J. Path. Bact.*, **68**, 547.—(1956) *Acta Un. int. Cancr.*, **12**, 653.
MARCHANT, J.—(1957) *Brit. J. Cancer*, **11**, 452.—(1959) *Ibid.*, **13**, 106.
MÜHLBOCK, O.—(1956) *Advanc. Cancer Res.*, **4**, 371.